# Editorial: The gut-immune axis: a complex training ground impacting inflammatory pathologies

**DOI:** 10.3389/fimmu.2023.1274761

**Published:** 2023-08-23

**Authors:** Jose Garrido-Mesa, Julio Gálvez, Natividad Garrido-Mesa

**Affiliations:** ^1^ Department of Medical and Molecular Genetics, Faculty of Life Sciences and Medicine, King’s College London, London, United Kingdom; ^2^ Department of Pharmacology, Center for Biomedical Research (CIBM), University of Granada, Granada, Spain; ^3^ Instituto de Investigación Biosanitaria de Granada (ibs.GRANADA), Granada, Spain; ^4^ Centro de Investigación Biomédica en Red de Enfermedades Hepáticas y Digestivas (CIBEREHD), Instituto de Salud Carlos III, Madrid, Spain; ^5^ Department of Pharmacy, School of Life Sciences, Pharmacy and Chemistry, Kingston University, London, United Kingdom

**Keywords:** gut, inflammation, genetics, immune repertoire, microbiome

Recently, the role of the gut in the development and training of the immune system has been increasingly recognised. The gut-immune axis is key in the pathogenesis of immune mediated inflammatory diseases (IMIDs), bridging genetic susceptibility and environmental factors. However, the specific mechanisms that connect changes in the gut and its microbiome with their impact at distant locations is far from being understood. Therefore, the identification of the genetic and environmental factors that operate through this axis and the mechanisms that predispose or protect from disease is an active area of research, addressed by several articles in this Research Topic. Elucidating these pathways and the alterations that lead to complex immune pathologies is essential to define their aetiology, progression and avenues for successful therapeutic intervention.

In this topic, research by Ma et al. shows that autoimmunity can predispose to leaky gut, as treatment with a TLR7/8 agonist led to decreased gut barrier integrity in lupus prone mice, but not in congenic healthy controls. This was linked to a reduced frequency in NKp46^+^ cells, critical for gut barrier integrity maintenance. Reduced intestinal permeability may secondarily lead to exacerbation of autoimmunity, by enhancing immune reactivity or inducing changes in the microbiome (dysbiosis).

The potential of studying the microbiota composition as diagnostic tool for altered gut-immune axis related pathologies is supported by Zhou et al. in the context of primary biliary cholangitis (PBC). Their study identifies six amplicon sequences as optimal biomarkers of PBC (Serratia, Oscillospirales, Ruminococcaceae, Faecalibacterium, Sutterellaceae, and Coprococcus), and functionally associates dysbiotic changes with altered lipid metabolism, highlighting the implications of this mechanism in PBC pathology.

Dysbiosis underlies the pathogenesis of multiple conditions, which can be explained by the microbiome as operator of genetic susceptibility. Cross-study analysis using bioinformatic tools, such as mendelian randomization (MR), and public datasets pairing GWAS and microbiome profiling allow to identify causal relations: i.e. genetic loci influencing a differential bacterial abundance that confers risk to a particular disease, whilst excluding cofounding variables with pleiotropic effects. Here, Cao et al. used MR to highlight the causal role of microbiome changes as operators of Sjogren’s Syndrome susceptibility loci. Both positive and negatively correlated bacterial taxa were identified, as well as the microbiome-related genes ARAP3, NMUR1, TEC and SIRPD, thus highlighting targets for genetically triggered microbiome alterations.

Environmental factors, such as diet, can underlie dysbiosis and its disease association. Zhao et al. have evidenced this in a novel model of diet-induced gut inflammation through arecoline supplementation. In this model, increased susceptibility to intestinal permeability and inflammation is ascribed to arecoline influencing the host metabolism through modulation of the microbiota.

Likewise, immune modulation can also be achieved through dietary interventions, as shown by Lo Conte et al. in type-1 diabetes NOD mice, where an anti-inflammatory diet, enriched in inulin and omega 3-PUFA, protected from autoimmunity and improved the metabolic profile, leading to gut barrier integrity restoration and changes in the microbiome (namely, an increase of mucus-degrading bacteria such as Akkermansia muciniphila and Akkermansia glycaniphila). In this model, characterised by the generation of islet-reactive T cells, the anti-inflammatory diet induced an expansion of FoxP3^+^ regulatory T cells and IL- 10^+^ Tr1 cells at the expenses of effector Th1/Th17 cells in the intestine, pancreatic lymph nodes and intra-islet lymphocytes. This study also provides proof of concept of gut-driven extra-intestinal pathology.

Importantly, dietary-induced immunomodulation is also possible without involving the microbiome. The gut immune system and microbiome composition varies along its longitudinal axis, with the colon being the primary site of bacterial colonization and the small intestine (SI) standing out for its nutritional role. However, the SI potential to modulate the immune system is not withheld. This anatomical location is the focus of the review by Bodmer et al. The authors describe its tolerogenic role sensing luminal contents and highlight the potential of SI targeted therapies, which induce atypical Tregs generation without altering the microbiome and/or compromising immunity to pathogens, an approach that could be exploited therapeutically to resolve auto-inflammatory pathologies.

Evidence of the gut-immune axis driving extra-intestinal pathology is also provided by He et al., who explain the impact of intestinal inflammation in neurological function through genetically-determined alteration of this axis. By MR analysis, they investigated inflammatory bowel disease genetic signature leading to morphological changes in the cerebral cortex, which may trigger neuropsychiatric disorders. They identified an interconnected network highlighting the top 10 variant-matched genes (STAT3, FOS, NFKB1, JAK2, STAT4, TYK2, SMAD3, IL12B, MYC, and CCL2) involved in neuroinflammation-induced damage, impaired neurological function, and persistent nociceptive input, ultimately leading to cortical reshaping.

Considering the breath of connections of the gut-immune axis, addressing the specificity of regulatory pathways is crucial to prevent unwanted off-site effects. In this sense, Peng et al. have performed an up-to-date revision of the role of the nuclear factor erythroid 2-related factor 2 (Nrf2) in ulcerative colitis (UC). The Nrf2 pathway regulates the intestine’s development and function and influences oxidant stress and inflammatory responses, being involved in the development of UC and UC-related intestinal fibrosis and carcinogenesis. The relevance of specific signalling pathways is also highlighted in Sun et al.‘s revision in the context of knee osteoarthritis (OA), also influenced by disturbances in the host-microbiome equilibrium. Here, they advocate for the identification of the signalling pathways activated by defined pathogens or specific microbiome changes as priorities for future research.

Targeted approaches like these, beyond the detection of intestinal permeability and dysbiosis underlaying multiple conditions, would help identify tissue/disease-specific alterations and their mechanisms in connection with the gut-immune axis, which will ultimately inform targeted therapeutic interventions. As these progress, future studies may be able to connect disease-associated changes in specific bacterial species with the immune repertoire, identifying species carrying bacterial/human cross-reactive epitopes as well as clonal populations associated with autoimmunity. Thus, whilst brief, this series of articles show the potential of interdisciplinary research to advance our understanding of the gut-immune axis, which warrants exciting discoveries ahead.

## Author contributions

JG-M: Conceptualization, Writing – original draft, Writing – review & editing. NG-M: Writing – review & editing. JG: Writing – review & editing.

